# Levels of cerebrospinal fluid α-synuclein oligomers are increased in Parkinson’s disease with dementia and dementia with Lewy bodies compared to Alzheimer’s disease

**DOI:** 10.1186/alzrt255

**Published:** 2014-05-07

**Authors:** Oskar Hansson, Sara Hall, Annika Öhrfelt, Henrik Zetterberg, Kaj Blennow, Lennart Minthon, Katarina Nägga, Elisabet Londos, Shiji Varghese, Nour K Majbour, Abdulmonem Al-Hayani, Omar MA El-Agnaf

**Affiliations:** 1 Department of Clinical Sciences, Lund University, Lund, Sweden; 2 Memory clinic, Skåne University Hospital, Lund, Sweden; 3 Neurology clinic, Skåne University Hospital, Lund, Sweden; 4 Institute of Neuroscience and Physiology, Department of Psychiatry and Neurochemistry, The Sahlgrenska Academy at University of Gothenburg, Gothenburg, Sweden; 5 Department of Biochemistry, College of Medicine and Health Sciences, United Arab Emirates University, Al Ain, United Arab Emirates; 6 Department of Anatomy, Faculty of Medicine, King Abdulaziz University, Jeddah, Saudi Arabia; 7 Faculty of Medicine, King Abdulaziz University, Jeddah, Saudi Arabia

## Abstract

**Introduction:**

The objective was to study whether α-synuclein oligomers are altered in the cerebrospinal fluid (CSF) of patients with dementia, including Parkinson disease with dementia (PDD), dementia with Lewy bodies (DLB), and Alzheimer disease (AD), compared with age-matched controls.

**Methods:**

In total, 247 CSF samples were assessed in this study, including 71 patients with DLB, 30 patients with PDD, 48 patients with AD, and 98 healthy age-matched controls. Both total and oligomeric α-synuclein levels were evaluated by using well-established immunoassays.

**Results:**

The levels of α-synuclein oligomers in the CSF were increased in patients with PDD compared with the controls (*P* < 0.05), but not in patients with DLB compared with controls. Interestingly, the levels of α-synuclein oligomers in the CSF were also significantly higher in patients with PDD (*P* < 0.01) and DLB (*P* < 0.05) compared with patients with AD. The levels of CSF α-synuclein oligomers and the ratio of oligomeric/total-α-synuclein could distinguish DLB or PDD patients from AD patients, with areas under the curves (AUCs) of 0.64 and 0.75, respectively. In addition, total-α-synuclein alone could distinguish DLB or PDD patients from AD patients, with an AUC of 0.80.

**Conclusions:**

The levels of α-synuclein oligomers were increased in the CSF from α-synucleinopathy patients with dementia compared with AD cases.

## Introduction

Alzheimer disease (AD) is the most common form of dementia, and with an increasingly aged population, AD is predicted to increase worldwide, causing suffering for the patients and their families and a large cost for society [1-3]. Other relatively common neurodegenerative disorders that cause dementia are dementia with Lewy bodies (DLB) and Parkinson disease with dementia (PDD). The symptoms and neuropathologies of these dementia disorders overlap to some extent. AD is characterized by the accumulation of intraneuronal depositions of hyperphosphorylated tau (neurofibrillary tangles) and extracellular aggregates of β-amyloid (amyloid plaques) [3]. DLB and PDD, however, are α-synucleinopathies that are characterized by intraneuronal aggregates consisting mainly of α-synuclein fibrils, which are found in Lewy bodies (LBs) and Lewy neuritis [4]. However, amyloid accumulation often also occurs in patients with DLB, and an AD-like pathology can also be found in patients with PDD. In addition, many AD cases also develop LBs [5,6].

The oligomerization of β-amyloid and α-synuclein appear to be key events in the pathology of AD and DLB/PDD, respectively [4]. Currently, several ongoing studies are addressing potential disease-modifying treatments that are directed against pathology-specific mechanisms, such as the aggregation and formation of the neurotoxic oligomeric species of β-amyloid or α-synuclein [7]. Biomarkers that can determine which brain pathologies underlie the symptoms of an individual patient, instead of classifying patients according to clinical syndromes, will be very helpful when selecting patients with early symptoms for new clinical trials to evaluate new disease-modifying therapies.

Biomarkers are available to aid in the diagnosis of AD, and several studies have shown that β-amyloid1-42 (Aβ1-42) level is decreased and that the total tau (t-tau) and phosphorylated tau (p-tau) levels are increased in the cerebrospinal fluid (CSF) of patients with AD compared with cognitively healthy controls [8-11].

Some studies have shown that the total levels of α-synuclein in the CSF are significantly decreased in patients with PD or DLB compared with patients with AD [12-14], but other groups have reported conflicting results [15-17]. During recent years, it has become increasingly evident that early aggregates or “soluble oligomers” of α-synuclein play an important role in the pathogenesis of α-synucleinopathies rather than the late aggregates or “amyloid fibrils.” Thus, high levels of soluble α-synuclein oligomers are present in the brain homogenates of patients with PD and DLB [18,19]. More recent studies have shown that the oligomeric forms of α-synuclein are neurotoxic *in vitro* and *in vivo*[20-23]. We and others recently reported elevated levels of α-synuclein oligomers and an increased oligomer/total-α-synuclein ratio in the CSF of PD patients compared with controls [24,25]. These findings suggested that CSF α-synuclein oligomers could be useful biomarkers for the diagnosis and early detection of PD [24,25].

We studied the levels of CSF α-synuclein oligomers in dementia cases with LBs compared with the levels in dementia cases with AD and in healthy elderly controls.

## Methods

### Study participants

In the present study, we included 247 CSF samples from subjects with AD (*n* = 48), PDD (*n* = 30), and DLB (*n* = 71) and from nondemented elderly controls (*n* = 98) at the Memory Clinic, Skåne University Hospital, Sweden. All patients underwent brain imaging; routine laboratory testing; and detailed neurologic, psychiatric and cognitive examinations by a medical doctor experienced in dementia disorders. Patients diagnosed with PDD met the Clinical Diagnostic Criteria for Dementia Associated with PD according to Emre *et al.*[26]. Patients who received an AD diagnosis met the DSM-IIIR criteria for dementia [27]. The criteria for probable AD were defined by NINCDS-ADRDA [28]. Patients with DLB met the consensus criteria according to McKeith *et al*. [29]. All controls underwent cognitive testing and neurologic examination by a medical doctor, and individuals with objective cognitive or parkinsonian symptoms were not included as controls in the present study.

All individuals gave informed consent either by use of a passive-consent procedure in which consent for the retrospective use of banked clinical samples and data was assumed if individuals did not actively retract permission, as instructed in local press advertisements, or by active written informed consent. This study procedure was approved by the local ethics committee at Lund University Sweden and conducted according to the Helsinki Declaration.

### CSF samples

The CSF samples were obtained by lumbar puncture in the L3/L4 or L4/L5 interspace in the morning from nonfasted patients. The samples were collected in polypropylene tubes and gently mixed to avoid gradient effects. All samples were centrifuged within 30 minutes at 4°C at 2,000 *g* for 10 minutes to remove the cells and debris and then stored in aliquots at -80°C until the biochemical analysis.

### Immunoassay for measuring oligomeric α-synuclein in the CSF

A 384-well ELISA microplate was coated by overnight incubation at 4°C with 1 μg/ml mAb 211 (Santa Cruz Biotechnology, USA) in 200 m*M* NaHCO_3_, pH 9.6 (50 μl/well). The plate was washed with phosphate-buffered saline (PBS) containing 0.05% Tween-20 (PBST) and incubated with 100 μl/well of blocking buffer (PBS containing 2.5% gelatin and 0.05% Tween-20) for 2 hours at 37°C. After washing, 50 μl of the CSF samples (thawed on ice before Tween-20 was added to a final concentration of 0.05%) was added to each well, and then the plate was incubated at 37°C for another 3 hours. Biotinylated 211 diluted to 1 μg/ml in blocking buffer was added, and the plate was incubated at 37°C for 2 hours. The plate was washed and then incubated for 1 hour at 37°C with 50 μl/well of ExtrAvidin-Peroxidase (Sigma-Aldrich, Dorset, UK). After washing, the plate was incubated with 50 μl/well of an enhanced chemiluminescent substrate (SuperSignal ELISA Femto; Pierce Biotechnology, Rockford, IL, USA). Then the chemiluminescence in relative light units was immediately measured with a Victor^3^ 1420 (Wallac) microplate reader [30,31]. The samples were screened in a blind fashion and tested randomly. The case and control samples were run on a single plate to avoid plate-to-plate variations, and the results were confirmed with at least two independent experiments.

### Analysis of total levels of α-synuclein in CSF samples

The levels of total α-synuclein were quantified by using a newly developed bead-based xMAP technology assay, and these results were included in a previous report [14]. In brief, a monoclonal antibody (MAb), 9B6 IgG1, which recognizes a human-specific α-synuclein C-terminal epitope in exon 5, was used as the capture antibody. The antibody was covalently coupled to carboxylated beads (region 126). The MAb 4D8 IgG1, an antibody recognizing an N-terminal epitope in exon 3 of α-synuclein, was used as the detector in its biotinylated form. The bead assay was combined with bead-controlling heterophilic antibody interference (a specific MAb, bead 150) [32]. Heterophilic antibodies are a common problem in immunoassays [33] and have been used to exclude samples in plasma studies [34]. Although the problem of heterophilic antibodies has also been acknowledged in CSF studies [35], heterophilic antibodies were not observed in any of the 247 CSF samples analyzed herein, by using an arbitrary cut-off of an MFI of 150. The assays were analyzed on a Luminex 100IS instrument.

### Statistical analysis

The statistical analyses were conducted with SPSS for Windows, version 20.0 (SPSS Inc., Chicago, IL, USA). The correlation analyses were performed by using the Spearman rank correlation test (R_s_). To compare the demographic and CSF baseline data between groups, the Mann–Whitney *U* test was used for continuous variables, and the Pearson χ^2^ test was used for dichotomous variables.

## Results

### The levels of oligomeric and total α-synuclein in the CSF samples

Both total and oligomeric forms of α-synuclein were assessed in the CSF samples from 71 DLB patients, 30 PDD patients, 48 AD patients, and 98 healthy elderly controls. The demographic data are presented in Table 1.

**Table 1:** Demographic data and the levels of total and oligomeric α-synuclein and the oligomer/t-α-synuclein ratio in the CSF

	**Controls**	**PDD**	**DLB**	**AD**
**Number**	**98**	**30**	**71**	**48**
**Male/Female**	**35/63**	**22/8**^a^	**49/22**^a^	**13/35**^c, d^
**Age, years**	**69** (11.6)	**76** (5.5)^a^	**74** (7.4)^b^	**77** (5.6)^a, e^
**MMSE**	**29** (28–30)	**23.5** (17–25)^a^	**21** (17–24.75)^a^	**21** (19–23)^a^
**α-Synuclein oligomers**	**37,882 (21,763–136,685)**	**73,309 (36,361–326,297)**^b^	**40,440 (22,235–137,845)**	**26,441 (21,548–44,332)**^b, c, e^
**α-Synuclein oligomers/α-Synuclein total**	**549 (372–2149)**	**1,333 (492–5,973)**^b^	**811 (379–2,238)**	**333 (199–718)**^a, c, d^
**α-Synuclein total**	**67.00 (52.00–86.00)**	**61.50 (51.25–68.25)**	**58.00 (43.75–75.00)**	**94.00 (76.00–121.00)**^a, c, d^

The data are presented as the median (25th to 75th percentile), except for age, where the data were normally distributed. The data for age are presented as the mean (SD). The α-synuclein oligomer levels are presented in RLU. The total α-synuclein levels are presented in ng/L. For α-synuclein total and α-synuclein oligomers/α-synuclein total, only CSF samples with hemoglobin less than 1,000 ng/L were included.

^a,b^Compared with the controls, ^a^*P* < 0.001, ^b^*P* < 0.05.

^c^Compared with PDD patients, ^c^*P* < 0.001.

^d, e^Compared with DLB patients, ^d^*P* < 0.001, ^e^*P* < 0.05.

**Figure 1:**
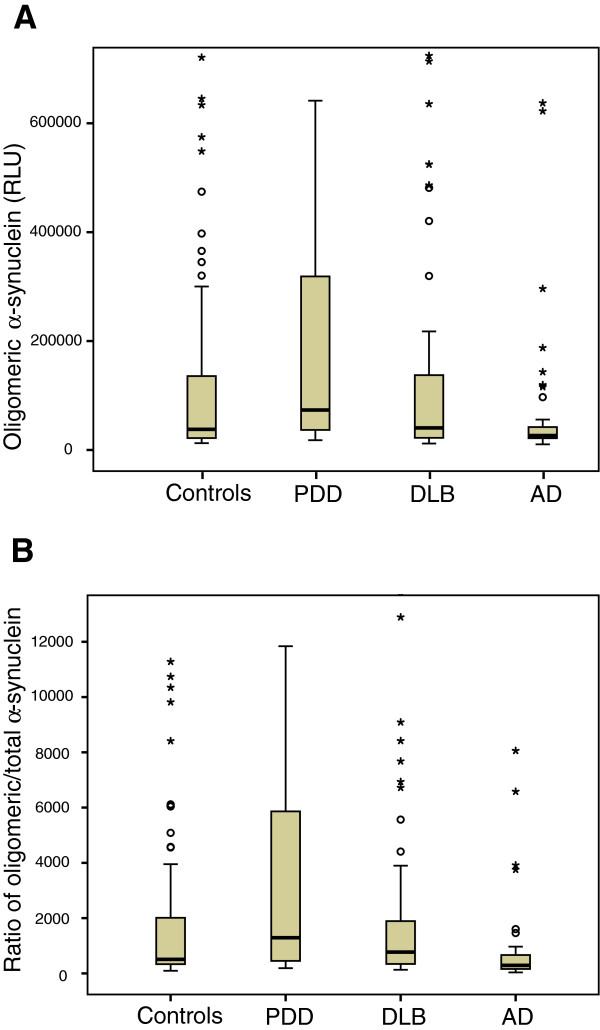
**Levels of α-synuclein oligomers (A; RLU, relative luminescence units) and the ratio of α-synuclein oligomers to total-α-synuclein (B; oligomer/total ratio, %) in the CSF of healthy elderly individuals (*n*** **= 98) and of patients with PDD (*n*** **= 30), DLB (n = 71) or AD (*n*** **= 48).** The levels of α-synuclein oligomers and the ratio were increased in patients with PDD compared with patients with AD and healthy controls (Mann–Whitney *U* test; *P* < 0.05). The box represents the interquartile range (IQR), with the median indicated in the middle. The error bars represent the lowest and highest normal values (max 1.5 box lengths from the lower and upper quartiles, respectively).

The levels of α-synuclein oligomers in the CSF were increased in patients with PDD compared with the controls (*P* < 0.05; see Table 1 and Figure 1), but not in patients with DLB compared with controls. Interestingly, the CSF levels of the α-synuclein oligomers were also higher in both the PDD and DLB patients compared with the AD cases (*P* < 0.01 and *P* < 0.05, respectively; see Table 1 and Figure 1A). Similarly, the ratio of α-synuclein oligomers/total-α-synuclein was also elevated in patients with PDD and DLB compared with the ratio in patients with AD (*P* < 0.01; see Table 1 and Figure 1B).

### Diagnostic accuracy of the oligomeric and total α-synuclein

**Figure 2:**
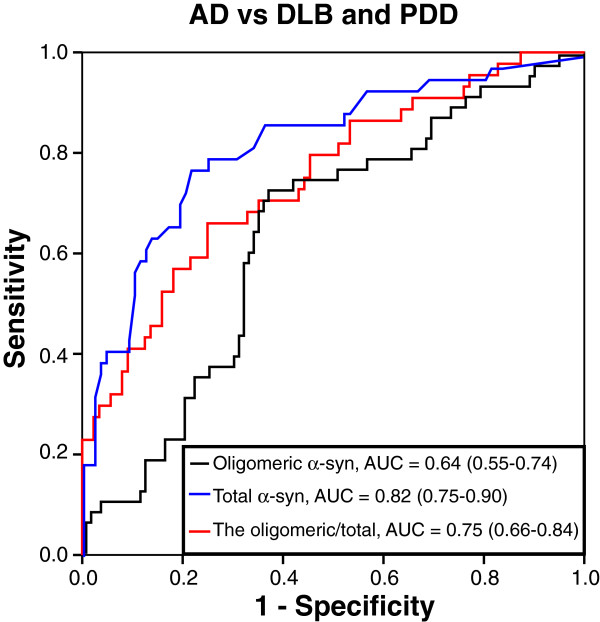
Receiver operating characteristic curves (ROC) showing the diagnostic accuracy of α-synuclein oligomers levels (black), the ratio of α-synuclein oligomers/total-α-synuclein (red) and total-α-synuclein levels (blue) when differentiating patients with DLB and PDD from patients with AD.

Both CSF α-synuclein oligomers levels and the α-synuclein oligomers/total-α-synuclein ratio could distinguish DLB and PDD patients from AD patients, with AUCs of 0.64 and 0.75, respectively. However, in this cohort, the diagnostic accuracy of the CSF total α-synuclein levels was even higher, with an AUC of 0.80 (Figure 2).

### Associations between oligomeric α-synuclein and cognitive performance

No significant correlations were found between the CSF α-synuclein oligomers levels and the cognitive performance, as measured with the MMSE in the PDD patients, DLB patients, and healthy controls. However, in the AD cases, increased levels of α-synuclein oligomers in the CSF correlated with worse performance on the MMSE (R_s_ = -0.31; *P* < 0.05).

No correlations appeared between the CSF α-synuclein oligomers and either age, gender, or disease duration (data not shown).

## Discussion

The discovery of missense and multiplication mutations in *SNCA* that were linked to clinical and pathologic phenotypes ranging from PD to PDD and DLB [36-39] highlighted the direct role of α-synuclein overexpression in the pathogenesis of these disorders. Furthermore, abnormal aggregates of α-synuclein protein were identified as the main components of LBs, the pathologic hallmark of PD, PDD, and DLB [40]. Therefore, α-synuclein misfolding and aggregation in the brain are considered pivotal factors in the degeneration process. Early aggregates or “soluble oligomers” of α-synuclein may be the pathogenic species that lead to neuronal death and neurodegeneration, rather than the late aggregates “amyloid fibrils” [21,22]. High levels of α-synuclein oligomers are present in brain homogenates from patients with PD and DLB compared with normal brains [18,19]. Interestingly, we and others previously reported significant differences between the CSF α-synuclein oligomers in PD patients compared with age-matched controls, with most of the PD samples showing higher levels of CSF α-synuclein oligomers than did age-matched controls [24,25]. The aim of this study was to determine whether α-synuclein oligomers levels and α-synuclein oligomers/total-α-synuclein ratio in the CSF are elevated in α-synucleinopathies cases with dementia compared with AD patients and elderly healthy controls. In the present study, we reported CSF levels of the oligomeric α-synuclein in PDD, DLB, and AD cases. Interestingly, we observed high levels of CSF α-synuclein oligomers and high α-synuclein oligomers/total-α-synuclein ratio in PDD and DLB, which was evident when compared with CSF samples from patients with AD (Figure 1). We found no associations between the CSF levels of the oligomeric α-synuclein and cognitive performance in patients with PDD or DLB.

In contrast, AD patients with higher levels of CSF α-synuclein oligomers exhibited a worse cognitive performance, although the correlation was very weak, indicating that AD patients with LB pathology could develop more severe dementia.

Currently, distinguishing dementia patients with AD from those patients with DLB relies on a clinical history and examination. However, to design a better treatment plan, objective methods to discriminate AD cases from DLB cases are needed. For example, the neuroleptic drugs that are often used to treat the psychiatric symptoms in AD can be detrimental to DLB patients. Because of the overlapping pathologies between these two disorders, the standard CSF biomarkers for AD (Aβ_1–42_, T-tau, and P-tau) do not readily discriminate between them [41-44]. Future large clinical studies are needed to evaluate whether CSF α-synuclein oligomers, when combined with biomarkers for AD, could increase the diagnostic precision in distinguishing dementia patients with AD from those patients with DLB and PDD.

We believe that the α-synuclein oligomers detected in CSF are derived from the neurons of the central nervous system. Therefore, the concentration of the oligomeric forms of α-synuclein in the CSF would correlate with the levels of soluble α-synuclein aggregates that are present in the brain. To address this issue, CSF studies with neuropathologic outcomes are needed. CSF α-synuclein oligomers might, however, serve as markers for selecting the correct patient population for clinical trials that are designed to evaluate new experimental therapies targeting α-synuclein oligomers in the brain. Selected patients with high levels of CSF α-synuclein oligomers could be more likely to respond to such therapies [7]. Moreover, the quantification of the levels of CSF α-synuclein oligomers at the baseline and during the treatment would assist with the identification of the most promising and effective drug candidates and doses in large-scale clinical trials.

## Conclusions

In summary, our results demonstrated that the levels of α-synuclein oligomers and oligomers/total-α-synuclein ratio in the CSF are increased in patients with dementia and LBs pathology. However, our findings need further validation by independent studies in independent cohorts with neuropathlogical outcome data.

## Abbreviations

AD: Alzheimer disease; CSF: cerebrospinal fluid; DLB: dementia with Lewy bodies; LBs: Lewy bodies; PDD: Parkinson disease with dementia; p-tau: phosphorylated tau; t-tau: total tau.

## Competing interests

The authors declare that they have no competing interests.

## Authors’ contributions

OH, SH, and OE performed the study design, interpretation of the results, and writing of the manuscript draft. AO, HZ, KB, LM, KN, EL, AH, SV, and NM contributed to the study concept and design, and to critical revision of the manuscript for important intellectual content. HZ, KB, LM, KN, and EL collected data and contributed to interpretation of results. SV and NM performed the experiments and analyzed and compiled data. OH and OE performed the study supervision. All authors read and approved the final manuscript.
